# From invention to progress: Energy technology innovation and sustainable development in OECD economies

**DOI:** 10.1371/journal.pone.0310104

**Published:** 2025-02-13

**Authors:** Khatib Ahmad Khan, Waheed Ahmad, Azeem Oluwaseyi Zubair, Mohammad Subhan, Muhammad Ibrahim Shah

**Affiliations:** 1 School of Business, Xi’an International University, Xi’an, China; 2 Glocal School of Business and Commerce, Glocal University, Saharanpur, UP, India; 3 Department of Economics and Quantitative Methods, University of Management and Technology, Lahore, Pakistan; 4 Department of Training and Workshop, One-to-One Research Institute and Consulting Ltd./Gte., Lagos, Nigeria; 5 Department of Commerce, Aligarh Muslim University, Aligarh, India; 6 Independent Researcher, Dhaka, Bangladesh; Damascus University, SYRIAN ARAB REPUBLIC

## Abstract

In the era of Industry 4.0, the advancement in energy technology has taken centre stage to mitigate climate change and promote sustainable development. Ever since the adoption of the United Nations SDGs in 2015, different regions and countries have been moving to achieve these targets by implementing various mechanisms. The OECD is one such region where aggressive funding towards equipment with high energy efficiency and the advancement of technologies for producing and consuming renewable energy are provided to advance towards sustainable development. Given the economic significance of the aforementioned countries, this study evaluates the influence of energy technology innovation on sustainable development in OECD countries. Our research focuses on energy technology innovation, which we measure through the energy technology R&D budget. In addition to energy technology innovation, we consider several other control variables such as state fragility index, financial development and foreign direct investment. In order to achieve the aforementioned goal, we utilize advanced econometric modelling methods of the second generation. These techniques encompass a CSD test, unit root tests, cointegration test, and CS-ARDL model. The result from CS-ARDL suggests that energy technology innovation enhances sustainable development in the short and long run. State fragility is shown to influence sustainable development negatively and significantly. The role of financial development as well as foreign direct investment, is found to be favourable for sustainable development. Based on the outcome, it is recommended that countries of this region significantly increase investment in energy technology, enhance financial development and encourage foreign direct investment along with tackling the fragility of these nations to boost sustainable development.

## 1.0 Introduction

Technological innovation is increasingly recognised as a crucial factor to increase economic development, create employment opportunities, raise standard of living and societal well-being [1]. It is considered to be the economic function and via this economic function, new technologies are brought to consumption and production [2]. Its role is currently defined as the enabler of digital automation and digital transformation. Technological innovation is now increasingly used in energy industry to increase the solutions for clean energy or for the achievement of energy efficiency [3]. Technological innovation in the energy sector is important because the loss of the production of energy occurs mainly during the beginning stage of extraction, transformation, transmission, transportation, and final consumption [4]. But at the same time, the world is experiencing higher greenhouse gas emissions, undermine the goals targeted to be achieved within 2030.

The OECD is comprised of 38 member nations and is one of the most significant trade associations in the world. As of the end of 2021, the gross domestic product of OECD members is valued at $57.92 trillion, underscoring its significance in the world economy [5]. Regardless of the COVID-19 epidemic, the majority of OCED member countries attained outstanding economic growth in 2021, with the United States recording a GDP valued at US$22,996,100.00, Japan (US$4,937,421.88), Germany (US$4,223,116.21), and the UK (US$3,186,859.74) as the four largest economies among the OECD members. This can be linked with industrialization, as most OECD members are developed nations. It is, however, expected that the combined value of GDP in the OECD will continue with its steady growth. However, the socioeconomic consequence of such value-added change on the environment should also be noted. The continuous utilization of innovative technology for final output will spur sustainable development goal’s objectives for 2030 and beyond.

**Fig 1:**
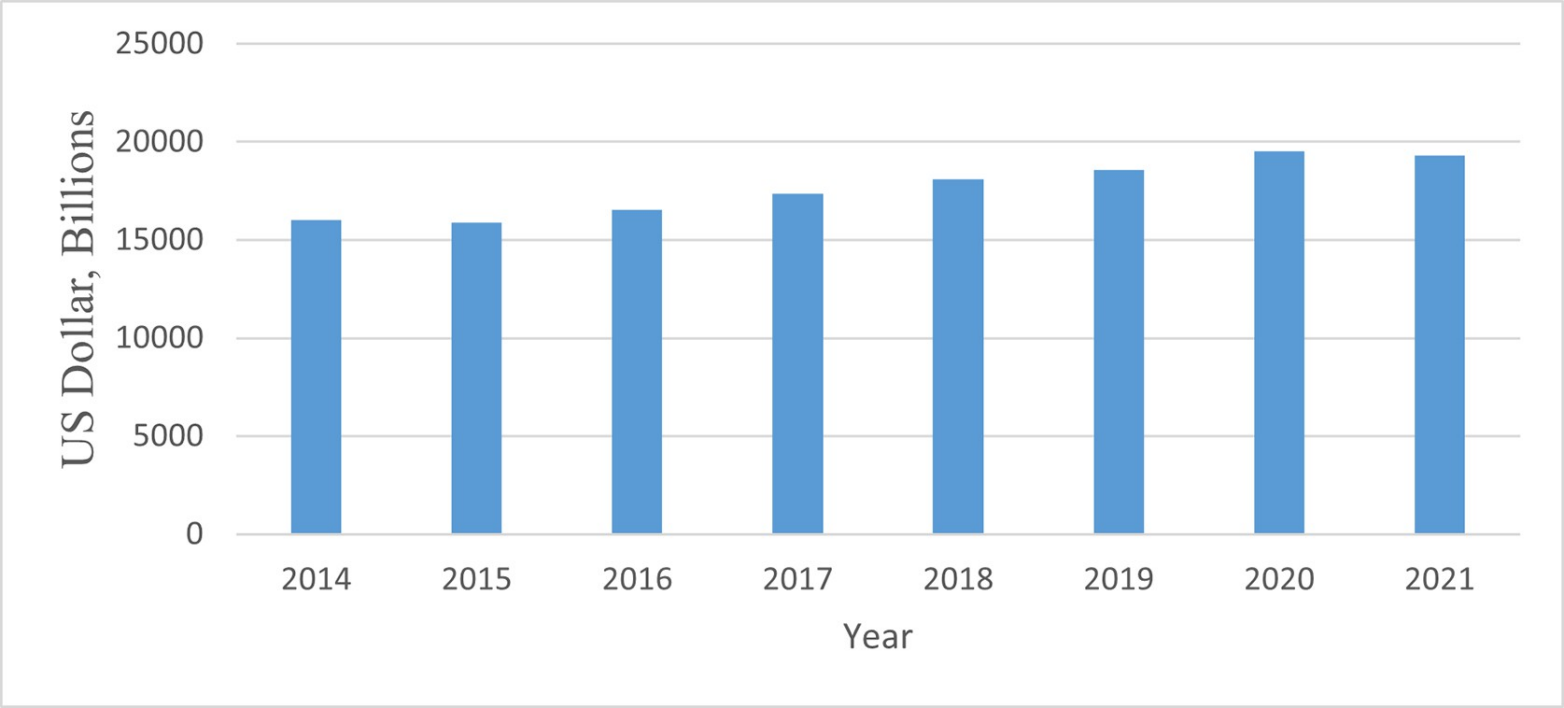
Government R&D budget allocation for energy in OECD. Source: OECD [7].

Fig 1 provides insights into R&D performance in energy development, which relies heavily on government support. As of 2021, energy R&D budgets in the OECD are anticipated to be US$19.2 billion. This estimate represents a marked decrease of US$ 219.8 million, equivalent to about 1.2% over 2020. However, R&D budgets increased by 4.85% between 2019 and 2020. Such a rise could result from increased R&D capital approved before Covid-19. Unfortunately, some resources and fiscal allocators lack comprehensive information about financial support for R&D for energy growth at such a crucial juncture. According to Curristine et al. [6], since the 1990s, the OECD government has carried out reforms to contain the growth in community spending to ensure a budget is responsive to priorities. However, the UN SDG on innovation is one of several priorities that the OCED government must achieve.

**Fig 2:**
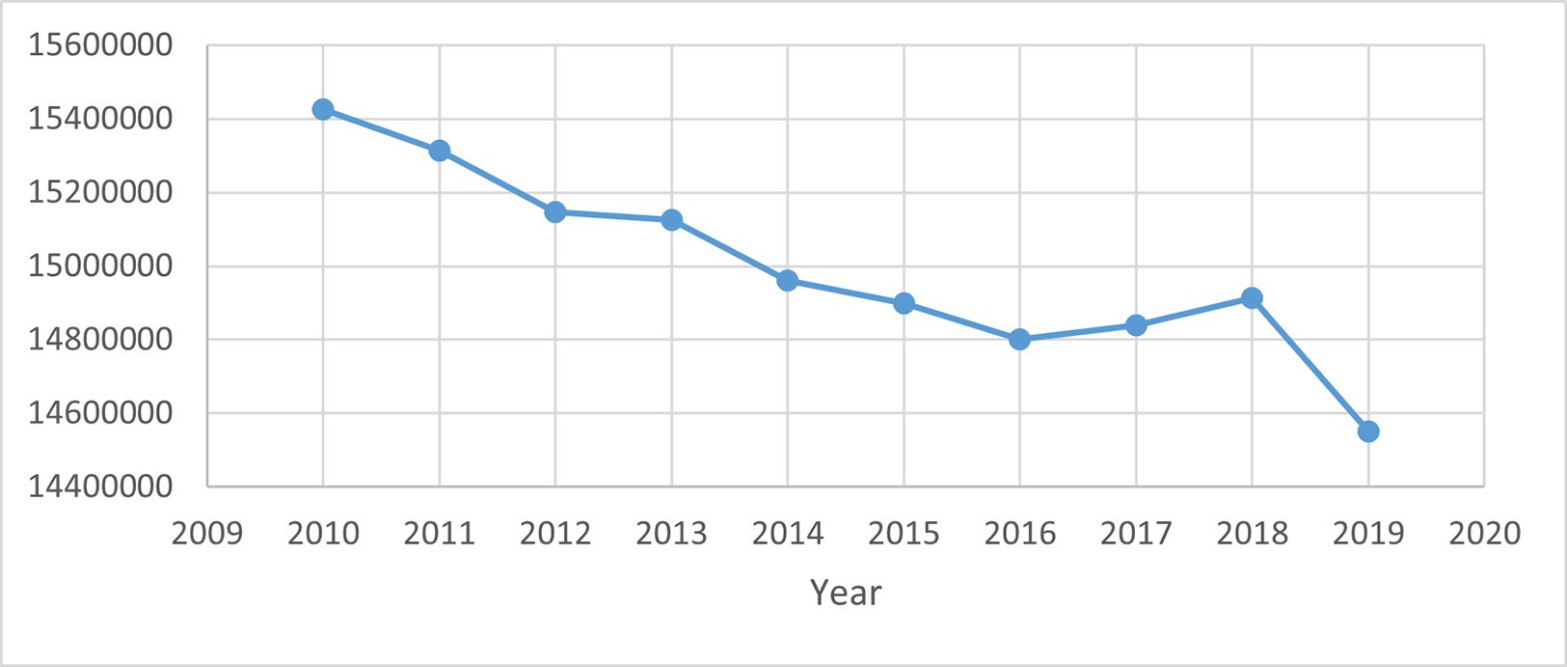
Total greenhouse gas emissions in OECD (kt of CO2 equivalent). Source: WDI [5].

Concerning environmental protection, the 2015 Paris Agreement specified the objective of reducing global warming to 1.5°C above pre-industrial levels while keeping global warming well below 2°C above pre-industrial levels, reiterated at the just finished 2022 COP27 in Egypt [8]. However, the roads to reaching the SDGs were irregular in the past, owing to a lack of sufficient financing, the volatility of fossil fuel costs, and political uncertainty [9]. In this regard, governments should enable more profound and more effective exchange of advanced technological knowledge [10]. Fig 2. displays the environmental scenario of the OECD from 2010–2019. The graph shows that the OECD maintains a consistent downward trend of CO2 emissions until 2018, when the emission level rises. However, during this period, the OECD has considerably reduced CO2 emission intensity by 874519.82 kilotonnes from 2010 to 2019. Thus, it is critical to examine the impact of energy-innovation technology on long-term sustainable growth in OECD countries.

Several studies have found that progress in technology, finance, and other disciplines can boost green growth and SD. For instance, Noja et al. [11] observed that in the European Union region, energy innovations encourage SD. Li et al. [12] discovered that green technological innovation improves green growth in China. Ulucak [13] examined and found the same outcome for China but using energy innovation. This research seeks to determine whether these outcomes are valid, given that Energy R&D investment has witnessed sluggish growth for over 30 years. Renewable energy’s share of the total primary energy supply is insignificant for most OECD member countries and the OECD.

**Fig 3:**
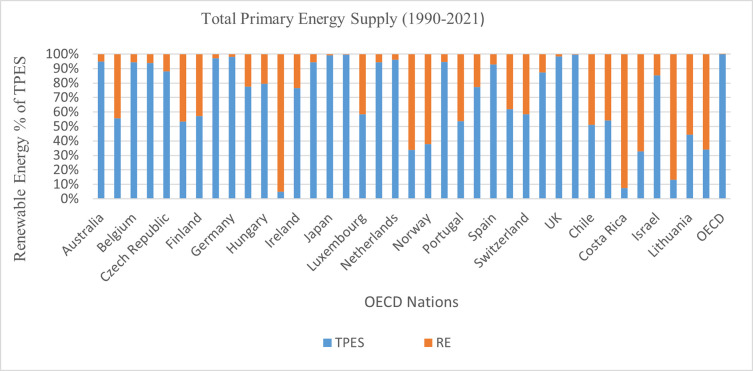
Primary energy supply and renewable energy in OECD. Source: OECD [19]; OECD [20].

**Fig 4:**
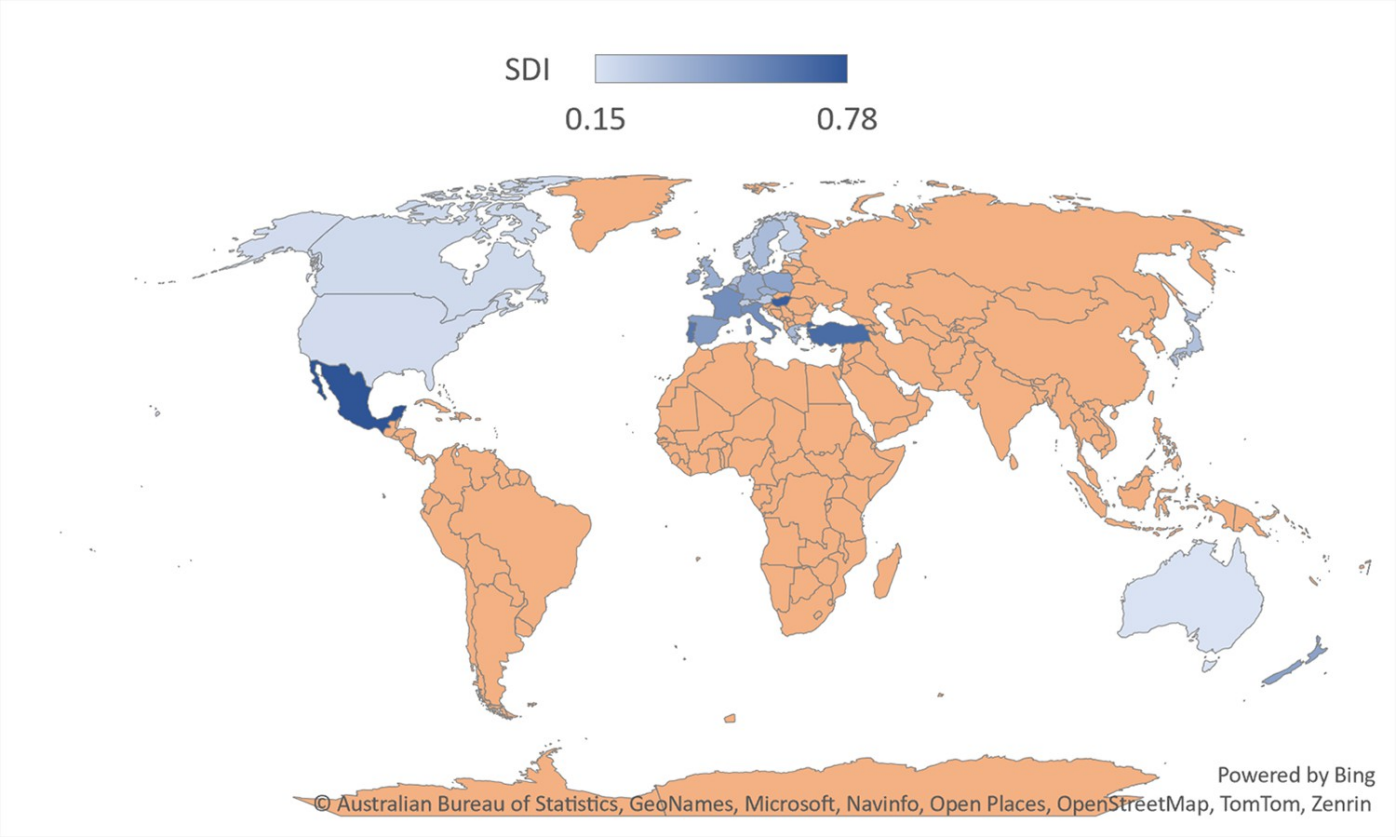
Map of sustainable development index in OECD countries. Source: Hickel [21].

Energy sector investment plays a key role in achieving sustainable development goals, Paris target and specific countries’ target as outlined in their nationally determined contributions. The SDG7 relates to this goal by providing cleaner, affordable, reliable and sustainable energy sources [14]. The modelling of Joint Research Centre (JRC) projects that this is the decade which will shape the way for the world to keep the 1.5-degree Celsius target possible. For this scenario to achieve, it is expected that energy sector’s investment will triple and reach more than 3 trillion USD, energy efficiency rate will double, and deployment of renewable will be 11 TW by 2030 [15]. For OECD to achieve their goals of energy policy, decrease emissions and develop a system of sustainable generation of electricity, it must invest more than 7.6 USD trillion over the next 25 years agenda [16]. Fig 4 indicates the share of renewable energy in the overall supply of primary energy (TPES) in OECD countries. Between 1990 and 2021, the average RE percentage of TPES in OCED is 7.45%. However, Iceland had the highest share (79.9%) of renewable energy percentage of TPES, followed by Costa Rica (46.5%), Norway (44.7%), New Zealand (33.7%), Latvia (30.9%), Sweden (30.9%), Chile (26.9%), Colombia (26.4%), Finland (24.7%), and Austria (24.5%). The lowest percentages were Korea (0.7%), Luxembourg (2.6%), the UK, Israel, the Netherlands, and Japan recorded a 3% renewable energy supply. It is worth noting that renewable energy supplies are more significant than other energy supplies in countries such as Iceland, New Zealand, Norway, Costa Rica, Latvia, Lithuania, and Slovenia. Meanwhile, renewable energy sources share a large percentage of TPES in countries such as Austria, Finland, Denmark, Luxembourg, Portugal, Sweden, Chile, and Colombia. Regarding the RES percentage of TPES, big economies such as the USA, UK, Japan, Korea, Poland, Italy, and Australia are far behind. Generally, the combined efforts of the OECD to increase renewable energy from the total primary energy supply are far from impressive (see Fig 3). The energy sector must significantly be accelerated by transitioning away from fossil fuel sector to low carbon solutions. This will be majorly enabled by the technological innovation specifically in the field of renewable energy. to improve socioeconomic and environmental outcomes, as energy consumption is essentially related to the development of the economy and environmental sustainability [17]. Therefore, in line with the global agenda, the OECD governments should reduce their subsidies for the fossil fuel industry, reduce barriers towards investment in green energy, encourage efficiency of energy sources and most important of all, energy innovation must be fostered which can target the gap of cost competitiveness. A revolution based on green energy will require all types of technology including carbon capture and storage, nuclear, smart grids and transport technologies as well as different types of renewable energy and energy efficiency. These will not only encourage security of energy sector but also provide enormous social and environmental advantages [18].

The OECD initiatives to boost member countries’ energy-innovation capabilities and to emphasize R&D activities and rewards that support the dissemination and acceptance of green technology prompted us to conduct this study. Thus, the research presented here extends to the existing literature on sustainable development focusing specifically on energy sector innovation. This study is unique in several contexts. For example, in contrast to previous literature, which mainly use greenhouse gas emissions, CO2 emission or growth as proxy for sustainable development, this research uses a novel measure of sustainable development developed by Hickel [21]. This measure includes both ecological impacts which is comprised of CO2 pollution and material footprint as well as development impact index which is comprised of education, life expectancy and income. Therefore, this research presents a comprehensive picture of sustainable development and how it is affected by energy sector innovation. Moreover, this research employs three other control variables specifically foreign direct investment, financial development and stage fragility.

In terms of methodologies, this study employs the heterogeneous CS-ARDL technique devised by Chudik and Pesaran [22], which estimates heterogeneous coefficients in a dynamic panel. During a regression, when dependence between cross-sectional units is not accounted for, CSD in error term occurs [23]. So, to capture cross-sectional correlation in the error term, the CS-ARDL model includes a linear combination of the cross-sectional means of the dependent variables and all the regressors. Chudik and Pesaran [22] argue that it is standard procedure to utilize both the Mean Group (MG) and Pool Mean Group (PMG) estimators when estimating the CS-ARDL model. Unlike other methods such as FMOLS, DOLS or GMM, this method has the capability to provide coefficients for both the short as well as long run. Other benefits of this method include the capability of incorporating I(1) and I(0) variables and its effectiveness when there is weak homogeneity arising from the lagged dependent variable. Furthermore, CCE-GMM by Neal [24] and CCE-MG by Pesaran [25] is used as a robustness analysis to handle endogeneity.

The remaining sections of this study are organized as follows: section 2 provides a literature survey, section 3 discusses research strategies and information sources, section 4 discusses and presents the expected findings, and section 5 addresses the summary and policy implications.

## 2.0 Literature review

### 2.1 Empirical literature review

The relationship between R&D and long-term economic growth is debated in development economics. Since the start of the twentieth century, academic recognition of technological innovation as a significant component in achieving sustainable development objectives has grown gradually [26–29].

Razzaq et al. [30] used region-based data from China to investigate how energy technology innovation in the renewable sector influences green growth. The authors used advanced quantile and generalized method of moments estimation from 2007–2019. They found that this type of innovation does not improve green growth in central and western regions. However, the authors showed that eastern region demonstrated more favourable outcome for this relationship. In another study for China by Li et al. [12], green technological innovation was used as a mediating factor in the association between green energy and green growth. The outcomes revealed that this type of technological innovation is directly and positively related to green growth. Furthermore, this innovation can improve the outcome of green energy on growth and thereby promote economic development which is sustainable. This innovation also works as a mediator regarding the effect of green energy on growth.

For the G7 nations, Wani et al. [31] tried to understand the linkage between green technology and green economic growth. They used several econometric techniques including CS-ARDL and CCEMG. They proved that green technology contributes towards green economic growth only during the long run, but it is insignificant during the short run. On the other hand, Ahmed et al. [32] discovered that green innovation is positively related to green economic growth for South Asia. In their analysis, they used data of 19 years and FMOLS and DOLS techniques. In another study for top countries of pollution, Ahmed et al. [33] found that eco technology or environmental technology has favourable impact on green growth.

While green growth has been subject of study in its relationship with green technology innovation, many empirical studies have focused on the relationship between technological innovation and environmental quality. In this regard, literature can be divided on the basis of the relation between general technological innovation and environmental quality as well as between green innovation and environmental quality. For example, Kihombo et al. [34] discovered that technological innovation reduces the ecological footprint in West Asia and the Middle East nations. A causality analysis further revealed that there is bidirectional causality between technological innovation and ecological footprint. In another study, Destek and Manga [35] reveals that technological innovation improvement effectively reduces carbon emissions although it does not have any significant impact on ecological footprint. Ahmad et al. [36] utilized the second-generation panel cointegration approach using data covering 1984 to 2016 to study the ecological footprint of 22 emerging economies. The results suggest that technological improvements are effective in minimizing the ecological harm caused by economic growth. On the other hand, Simeon et al. [37] utilized E7 countries to provide evidence that technological innovation helps to achieve net zero transition.

Now, Chu et al. [38] took 20 OECD countries to investigate the relation between environment related technology and ecological footprint. The study findings revealed that environmental technology reduces ecological footprint during the long run, but not during the short run. In another research, the impacts of energy innovation, globalization, and non-renewable energy on the ecological footprint of the G7 economies from 1990 to 2018 are investigated using CS-ARDL methodologies [39]. The findings suggest that energy innovation and globalization negatively impact the environment. The authors offered a policy agenda for the G7 nations focusing on SDGs 7, 8, and 13 based on the empirical results.

Altıntaş and Kassouri [40] utilized linear and nonlinear panel methods to study the influence of government energy technology RD&D on cleaner energy supply and carbon footprints in Europe from 1985 to 2016. According to the study, higher public financing for energy technology R&D reduces carbon footprints more than energy technology innovation promotes renewable energy adoption in Europe. Yıldırım et al. [41] use a similar method to analyze the effects of environmental innovations on energy sector-based carbon dioxide emissions in OECD countries. The researchers postulated that environmental innovations alone are not enough to address environmental concerns; they need to be backed by ecological policies to show their environmental reflections.

Noja et al. [11] investigated the impacts that advancements in digital technology, environmental performance, and energy innovations play in fostering more sustainable economic development in the European Union (EU) member states. Their findings demonstrate considerable positive benefits from energy breakthroughs, enhanced environmental performance, and digital transformation in the EU nations’ efforts to achieve sustainable development. Ulucak [13] also unravelled similar outcomes concerning the positive impact of energy innovation on environmental performance via budget for R&D in the energy sector in China. Using data period 1996 and 2017, Ahmad et al. [42] used the generalized method of moments (GMM) to evaluate the dynamic interaction linkages between sustainable energy investment, air pollution, and the sustainable development of 27 Chinese provinces and municipalities. According to the study, ecologically responsible energy expenditures reduce air pollution and boost sustainable development in China.

Taking carbon emission as a proxy for sustainable development, Fang [43] explored how energy sector investment and green technological innovation help to achieve sustainable development. The study was done for Chinese provinces considering more than 10 years of data. They found that China’s goal of carbon abatement can be achieved via these two mechanisms. Taking the same dependent vartiable, Sun and Razzaq [44] discovered that green innovation induces less emission in OECD. For the BRICS nations, Baloch et al. [45] investigated how energy innovation can advance sustainable development considering greenhouse gas emissions. Utilzing FMOLS and DOLS type regressions, this study provided evidence that energy innovation can decrease GHG emissions. In another recent study, Alnour et al. [46] took emission and growth separately as dependent variables and examined how energy technology innovation can affect them both. They considered 34 countries and 10 years of data. They found that general R&D expenditure cannot influence CO2 in the short run, and the long run shows the opposite impact. Furthermore, they found no evidence of a relationship between energy patents and the growth of the economy. In another study, Habiba et al. [47] revealed that green technology innovation promotes environmental quality by reducing emissions. The forecasting mechanism also showed this for future periods.

### 2.2 Research gap

While energy technology innovation has direct implications for the energy sector, there is a gap in exploring its invention to progress sustainable development in OECD economies. This research made efforts to provide a more nuanced and comprehensive understanding of the relationship between energy technology innovation and sustainable development in OECD economies. This research is important for the OECD since it consists of both developed and developing nations in continents and regions globally and can thus provide a finding that may not be like others. A large majority of OECD member countries are frontiers in terms of sustainable development and energy innovation objectives, making them an example to follow by the rest of the world. It is therefore worth noting that this research is unique with the consideration for geographical and contextual differences among these economies, which is expected to lead to varying innovation and sustainability outcomes. While energy innovation technology is used as the primary independent variable of this study, few studies modelled how financial development, foreign direct investment, and the fragility index alongside sustainable finance can affect sustainable development in OECD countries. Specifically, our measure of institutional quality by state fragility index is unique since the majority of the studies consider worldwide governance indicators as a measure of institutional quality.

Furthermore, the study is unique in terms of its use of methodologies. For example, to consider short term and long-term effects, the study considers CS-ARDL technique which can handle cross sectional dependence bias as well as slope heterogeneity [48]. On the other hand, the study considers CCE-GMM estimation by Neal [24] to take care of endogeneity. Most of the recent studies on energy innovation and development nexus do not account for endogeneity, which may produce biased results, and our study’s unique contribution lies there.

## 3.0 Data and methodology

### 3.1 Theoretical framework

The environmental sustainability of a nation is undermined when it relies heavily on non-renewable energy sources whose consumption and extraction processes pollute the environment. Therefore, researchers have suggested going for an energy transition, which posits that nations must transition away from non-renewable energy sources and adopt more renewable energy sources. The above statement is based on the energy transition hypothesis, which advocates going from low-efficient energy sources to more efficient ones [49]. On the other hand, there is green innovation theory, which describes the way economic growth can be sustainable via the adoption of environmental technologies as they can remove environmental externalities and, at the same time, enhance the efficiency of resources, all of which further increases sustainable development [50].

The continued investment in R&D accelerates the development and deployment of sustainable energy technologies and drives progress toward a cleaner, more resilient energy system. Meanwhile, adopting and scaling energy innovation can create new economic opportunities, promote job creation, and improve consumer energy affordability and environmental sustainability in OECD economies [18]. ENIN breakthroughs are critical because of their enormous consequences for environmental preservation and the progress of sustainable practices. Since climate warming is a major challenge all over the world, adopting new and progressive energy technology, such as renewable and energy-efficient systems, tackles climate concerns directly by lowering greenhouse gas emissions, which have increased during recent years [39, 51]. Furthermore, these advances support responsible resource utilization by maximizing energy output and limiting resource waste. Energy innovations strengthen the pillars of sustainability by guiding a nation toward cleaner and more resilient energy solutions, allowing the transition to a greener and more affluent future. Therefore, the first research hypothesis of this study can be formed as follows:

H1: Energy technology innovation promotes OECD countries’ sustainable development.

Along with energy, finance is also critical for social and economic progress [52]. Financial institutions such as banks are irreplaceable in financial as well as economic system. A well-developed financial sector assures effective capital allocation and promotes innovation [53]. This, in turn, can lead to the development of ecologically sustainable solutions, elevating a country’s SDI by promoting a symbiotic relationship between economic expansion and environmental consciousness [54]. Therefore, the second research hypothesis is as follows:

H2: Financial development favourably influences the sustainable development.

Foreign direct investment (FDI) is chosen as a variable because of its unique ability to create a holistic approach to sustainable development. FDI has a plethora of advantages, including the transfer of new technologies and production methods that are environmentally friendly [55]. Furthermore, FDI inflows frequently promote renewable energy transition and can provide technical and knowledge spillovers [56]. This transfer of advanced technology highlights FDI’s potential to act as a channel for sustainable economic development and boosting economic growth [57]. With regard to FDI, the third research hypothesis can be formed:

H3: Foreign direct investment has a positive influence on sustainable development.

Finally, state fragility is used to assess the vulnerability of states to conflict and instability. The index is helpful for policymakers and international organizations to identify and prioritize countries at risk of instability and design effective interventions to prevent conflict and promote development. It is expected that fragility will harm sustainable development and therefore, has negative effects. Hence, the final hypothesis of this study is as follows:

H4: State fragility undermines the OECD countries’ achievement of sustainable development goals

### 3.2 Data description

The current study evaluates how well nations are doing in terms of fulfilling sustainable development goals (i.e. Sustainable Development Index). This study’s primary goal is to ascertain how energy innovation (ENIN), state fragility index (SFGI), financial development (FIN), and foreign direct investment (FDI) affect sustainable development (SDI) in 28 OECD economies over the period 1990–2020. The study performed nearest neighbour interpolation to fill out the missing values following Cox [58] and orthogonal transformation has been conducted to take care of multicollinearity following Resch and Kock [59]. Fig 4. presents the map of the sustainable development index.

SDI is the dependent variable of this study, sourced from Hickel [21]. It is an efficiency indicator developed to evaluate nations’ effectiveness in terms of their contribution to human progress while minimizing their unfavourable impact on the environment. The calculation involves dividing a "development index", which is determined by the geometric mean of the “life expectancy index”, “education index”, and a modified income index, by an “ecological impact index”. The "ecological impact index" is determined by measuring the extent to which consumption-based CO2 emissions and material footprint surpass per-capita shares of planetary boundaries. Thus, the SDI can assist in identifying policies and practices that support sustainable development and motivate nations to take action toward a more sustainable future by monitoring progress over time in considering OECD economies.

Energy innovation is our focus variable. R&D per thousand USD in energy technology is a proxy of energy innovation. ENIN data is extracted from the International Energy Agency (IEA). Domestic credit provided by banks to the private sector is used as a measure of financial development (FIN) and it is sourced from WDI. Foreign direct investment (FDI) data is collected from WDI and the state of fragility index (SFGI) data is collected from the INSCR database for OECD economies. Table 1 provides the variable names, definitions, sources and prior expectations.

**Table 1:** Description of data.

Abbreviation	Indicator name	Source	Priori Expectation
SDI	Sustainable Development	Hickel (2020)	
ENIN	Energy Innovation	IEA	+
FIN	Financial Development	WDI	+
FDI	Foreign Direct Investment	WDI	+
SFGI	State Fragility Index	INSCR	-

Source: Authors’ own calculations

Energy innovation, financial development, FDI, and SFGI have been chosen to encapsulate the delicate fabric of sustainable development. This approach seeks to provide a complete and nuanced perspective on the several aspects that drive the Sustainable Development Index (SDI) by unravelling the delicate threads connecting economic growth, environmental stewardship, and societal advancement.

Our study introduces a model that depicts SDI as a function of ENIN, SFGI, FIN, and FDI. We follow the existing literature to analyse and compare the impacts of ENIN, SFGI, FIN, and FDI on SDI:

$${SDI}_{it}=\beta_{0t}+\beta_{1t}{ENIN}_{it}+\beta_{2t}{SFGI}_{it}+\beta_{3t}{FIN}_{it}+\beta_{4t}{FDI}_{it}+{ԑ}_{it}$$ (1)

Here, SDI refers to sustainable development, ENIN is energy innovation, SFGI is state fragility, FIN is financial development and FDI is foreign direct investment. *β* refers to the coefficients, ԑ is an error term, t refers to the time period, and i indicates the country of OECD.

For improved distribution, data is turned into a logarithmic shape [60]. The difficulties of the heteroscedasticity problem and autocorrelation in the data are also lessened by log form [61, 62]. As a result, the log-log form is as follows:

$${lnSDI}_{it}=\beta_{0t}+\beta_{1t}{lnENIN}_{it}+\beta_{2t}{lnSFGI}_{it}+\beta_{3t}{lnFIN}_{it}+\beta_{4t}{lnFDI}_{it}+{ԑ}_{it}$$ (2)

Here, ln refers to the logarithmic transformation.

### 3.2 Methodology

#### 3.2.1 Preliminary tests

The analysis’s data is built using panel settings. This sample has T greater than N. So, in this case, we used panel cointegration analysis. Fixed and random effect models are suitable for cases where the number of observations (N) is greater than the number of time periods (T), according to Zoundi [63]. Conversely, if the number of time periods (T) exceeds the number of observations (N), it is not advisable to use random and fixed effects models.

Panel data offers benefits for economic research, such as capturing individual and time-specific effects, analyzing dynamic relationships, and providing a larger sample size. However, Cross-Sectional Dependence (CSD) in residuals is a limitation that can result in biased and inefficient estimates. Our world today is marked by a globalized society where economies are interconnected and financially integrated. This interconnectedness has given rise to the CSD problem, as observed by De Hoyos and Sarafidis [64] in their research. Hence, the residuals of panel data are influenced by cross-sectional features due to neglected common factors, mutual disturbances, spatial influences, externalities, and unobserved cross-sectional dependence [65, 66]. To begin panel data analysis, it is essential to confirm the existence of CSD as a crucial step following CSD test of Chudk and Pesaran [23].

Due to difficulties with the size distortions and too often rejection of the null hypothesis, first-generation unit tests in CSD produce misleading results [67]. In the process of analyzing the panel variable for a unit root, it is essential to factor in the CSD. In contrast, the examination of the order of integration for variables in panel data involves the utilization of second-generation unit root tests, which take into account the CSD as noted by Pesaran [68]. By utilizing the second-generation unit root test we are able to examine the variable’s stationarity while accounting for cross-sectional dependence (CSD). Hence, it assists in deciding whether to employ second-generation or first-generation unit root tests. To achieve this, we utilize a range of unit root tests, such as the Pesaran [68] CIPS test and CADF tests. Recent research has shown that CIPS is becoming more popular for its ability to handle CSD and heterogeneity. The unit root series represents the null hypothesis of the test. Furthermore, the test suggests conducting a cointegration test before parameter estimation if the variables are stationarity is at the first difference.

Panel cointegration tests confirm long-term relationships between variables, and researchers can choose from various tests that have different advantages [69–71]. Despite the CSD’s existence, the Westerlund [71] ECM panel cointegration test can yield reliable results. Compared to Pedroni [70] residual-based cointegration test, the Westerlund [71] test has a strong ability to detect cointegration. Furthermore, Persyn and Westerlund [72] found that the analysis outcomes are affected by the duration of lead and lag in samples with a short temporal dimension. This test uses four distinct statistics, including panel statistics (Pt, Pa) and group statistics (Gt, Ga), to identify cointegrated cross-sectional units. The statistical measures of group mean (Gt, Ga) assume that all components of the cross-sectional data are cointegrated, while the coefficients of the long- and short-run ECM models may have different characteristics.

#### 3.2.2 Main techniques

We used the CS-ARDL model by Chudik and Pesaran [22] to analyze the connection between our variables. This model is an improved version of the panel ARDL-PMG by Pesaran et al. [73] and allows for a comprehensive examination of the variables’ relationship. The CS-ARDL framework includes short-term and long-term cross-sectional averages of relevant variables, parameters, error correction terms, and other components. This strategy has several advantages over alternative approaches. It can provide reliable estimates, even when variables are included in different orders, such as I (0) or I (1). Second, Chudik and Pesaran [22] showed that this method produces accurate results for both short-term and long-term CSD events. Our third point is derived from a collective estimation of a group, which encompasses a range of slope coefficients. The CS-ARDL model is an improved version of the ARDL model that incorporates average cross-sectional estimates for each cross-section.

Additionally, this approach is effective when the lagged dependent variable causes weak homogeneity. The following describes the regression’s basic model:

$${\Delta SDI}_{it}=c_{i}+{£}_{i}({SDI}_{it-1}‐\beta_{i}X_{it-1}‐{¥}_{1i}{\bar{SDI}}_{t-1}-{¥}_{2i}{\bar{X}}_{t-1})+\sum_{j=1}^{p-1}{Ω}_{ij}{\Delta SDI}_{it-j}+\sum_{j=0}^{p-1}\alpha_{ij}{\Delta X}_{it-j}+\pi_{1i}\Delta {\bar{SDI}}_{t}+\pi_{2i}\Delta {\bar{X}}_{t}+{ԑ}_{it}$$ (3)

Where Δ*SDI*_*it*_ is the dependent variable, independent variables in the long run estimates are denoted by X_it_, mean of the dependent variable in the long run is denoted as ${\bar{SDI}}_{t-1},{\bar{X}}_{t-1}$ denotes the mean of independent variables in the long run. Now, ΔSDI_it−j_ denotes the dependent variable for the short run and ΔX_it−j_ refer to the independent variables in the short run. Furthermore, ${\bar{\Delta SDI}}_{t}$ and $\Delta {\bar{X}}_{t}$ represent the average values of the dependent and explanatory variables in the short run. Further ԑ_*it*_ represents the random error terms. Additionally, the specification uses j for cross-section units and t for time. *β*_*j*_ represents independent variables coefficients for the long run, Ω_*ij*_ and *α*_*ij*_ represent estimated coefficients of dependent and independent variables for the short run and *π*_1*i*_ and *π*_2*i*_ represent the mean of dependent and independent variables in the short run.

To enhance the robustness of our analysis, we used additional estimation techniques alongside the CS-ARDL model. These included the CCE-GMM by Neal [24] and CCE-MG by Pesaran et al. [25] methods. By incorporating these checks, we ensure the reliability and validity of our findings, strengthening the credibility of our research. The additional advantage of the CCE-GMM estimator over CS-ARDL and CCE-MG is that it can handle the reverse causality between sustainable development and energy innovation. Sustainable development contains both the development and ecological indices. Higher development may ensure investment in energy technology, which can improve energy innovation. On the other hand, increased environmental degradation may also require the government to invest more in ENIN. Therefore, it is important to handle the reverse causality between SDI and ENIN by using the CCE-GMM estimator, where the 2^nd^ and 5^th^ lags of ENIN are used as instruments.

## 4.0 Empirical results

### 4.1 Descriptive statistics

The descriptive statistics for raw variables related to the economies considered to be OECD countries are displayed in Table 2. According to the descriptive analysis, the mean values of SDI and ENIN are less than 1, while those of SFGI, FIN and FDI are more than 1. Specifically, it can be seen that the value of FIN is the highest among all indicators, while that of ENIN is lower. The Table 2 presents the skewness and kurtosis, which also show several significant points; from the skewness statistics, it can be seen that the majority of the variables except for SDI show negative values, implying that the left tail is long compared to the right tail. However, for SDI, the right tail is long compared to the left tail.

**Table 2:** Descriptive statistics.

Variables	Mean	Std. Dev.	Min	Max	Skew.	Kurt.
SDI	0.494	0.181	0.150	0.795	-0.126	1.758
ENIN	0.296	0.278	0.002	2.596	2.605	15.409
SFGI	1.305	2.282	0.000	12.000	2.882	11.638
FIN	86.794	42.019	11.612	201.259	0.304	2.407
FDI	3.866	8.007	-41.508	86.589	4.118	35.16

Source: Authors’ own calculations

On the other hand, for a standard normal distribution, kurtosis is 3. Kurtosis greater than 3 are considered leptokurtic with long and thick tails and with more chances of outliers. The Table 2 shows that except for SDI and FIN, all other variables have leptokurtic distributions, meaning that there are higher chances of outliers as they have thick and very long tails. The platykurtic nature of SDI and FIN indicates that they have a thin tail and most of the series’ values are in the high proximity to the mean.

### 4.2 Preliminary tests

Checking the stationarity of the parameter estimation is essential before beginning the analysis, and unit root tests will be used to do this. Nevertheless, the unit root tests vary in terms of CSD among model parameters. The distinction between the first and second generations of the unit root test highlights the existing disparity. So, it is essential to comprehend the nature of CSD among the model parameters to select an appropriate unit root test. We used the cross-sectional dependence test by Chudik and Pesaran [22] to examine the weak CSD null hypothesis. The test result shown in Table 3 reveals that the model parameters have a substantial cross-sectional dependence. This particular piece of data supports the validity of the second-generation unit root testing. We used the CADF test and the CIPS test by Pesaran [68] to examine the stationarity property of the variables. The CIPS and CADF tests depict that ENIN and FDI are stationary at this level. The findings have been recorded in Table 4.

**Table 3:** CSD test.

Variables	Statistics	Variables	Statistics
lnSDI	65.077***	lnENIN	21.336***
lnSFGI	4.659***	lnFIN	30.811***
lnFDI	21.760***		

***, ** and * stands for 1%, 5% and 10% levels of significance individually

Source: Authors’ own calculations

**Table 4:** Second generation unit toot test.

Variables	CIPS		CADF	
	Level	First difference	Level	First difference
lnSDI	-1.763	-4.685***	-1.974	-3.689***
lnENIN	-2.875***	-	-2.669**	-
lnSFGI	-1.569	-3.276	-1.515	-2.73**
lnFIN	-1.729	-3.989***	-2.343	-2.591*
lnFDI	-3.865***	-	-2.712**	-

Note: ***, ** and * denotes 1%, 5% and 10% significance level. Source: Authors’ own calculations

On the contrary, SDI, FIN, and SFGI are stationery after the first difference. With the exception of SFGI, the unit root tests yield similar findings. The CIPS test revealed that SFGI had an undefined stationary level. Thus, these tests indicate the mixed integration order.

After Verifying the considered variables’ integration order or the stationarity level, we employed the Westerlund [71] test for the next step. Table 5 shows this result. The null hypothesis is rejected for pt and pa at a 1% significance level, conforming to cointegration. Based on the available evidence, it can be postulated that there is a long run association between the variables.

**Table 5:** Westerlund [71] cointegration test.

Statistic	Value	Z-value	P-value	Robust P-value
Gt	-0.844	5.839	1.000	1.000
Ga	-1.5	6.235	1.000	1.000
Pt	-3.629	3.888	1.000	0.000
Pa	-1.275	3.503	1.000	0.000

Source: Authors’ own calculations

### 4.3 Main results

After verifying the enduring relationship between the variables, we assessed the short and long-run effects of the variables on SDI using the CS-ARDL estimation technique. Table 6 shows the short-term and long-term outcomes. The error correction or adjustment term is -1.663 and significant, which is according to the expectations. However, this value is higher than 1, indicating that the process of the error correction does not monotonically and directly converge to the path of equilibrium, but the value in the long run experiences fluctuations that are subdued in nature [74]. However, as described by Narayan and Smith [75], convergence happens rapidly to the path of equilibrium after the completion of this process. The multicollinearity table in Table 7 shows that the mean VIF is 1.03, indicating no issue of multicollinearity in the model.

**Table 6:** Cross-Sectionally Augmented Autoregressive Distributive Lag (CS-ARDL).

Variables	Coef.	Std. Err.	P value
Short Run Est.			
L.lnSDI	-0.663	0.05	0.000
lnENIN	0.087	0.03	0.004
lnFIN	0.415	0.086	0.000
lnFDI	0.077	0.04	0.054
lnSFGI	-0.128	0.062	0.04
Long Run Est.			
lnENIN	0.05	0.017	0.004
lnFIN	0.24	0.049	0.000
lnFDI	0.049	0.026	0.059
lnSFGI	-0.065	0.032	0.041
Adjust. Term	-1.663	0.05	0.000

Source: Authors’ own calculations

**Table 7:** Test of multicollinearity.

Variable	VIF	1/VIF
lnENIN	1.01	0.995004
lnSFGI	1.05	0.951375
lnFIN	1.06	0.946852
lnFDI	1.00	1
Mean VIF	1.03	

Source: Authors’ own calculations

The findings illustrate that a rise in the ENIN level has a significant impact on the SDI within OECD economies. It indicates that a 1% increase in ENIN promotes the SDI to about 0.087% in the short run. The CS-ARDL’s long-run outcome revealed that a 1% increase in ENIN contributed to SDI by approximately 0.05% at a 1% significance level in the proposed OECD nations. Thus, the investment in energy innovation significantly enhances sustainability without compromising environmental degradation.

Omri et al. [76] stated that investing in RE can curb reliance on fossil fuels, mitigating harmful emissions and addressing climate change. Another study indicates that investing in renewable technologies can lower resource pollution consumption, reduce emissions, and achieve sustainable development [77]. The UNDP [78] report suggests that investments in energy infrastructure can increase energy availability in areas with unreliable access, supporting economic growth and elevating the quality of life. It may have the potential to generate jobs in the energy sector and adjacent industries, which can help with economic growth and raise the standard of living and could be able to achieve the 2030 SDGs agenda. Besides, the R&D funding can stimulate the development of new energy technologies and procedures that will aid in the transition to a low-carbon economy and sustainable development in OECD economies. Hence, the R&D in energy technology has significant repercussions for sustainable development in OECD members economies. The OECD acknowledges that technical innovation is essential to promoting economic success and environmental stewardship, and its priorities for fostering this innovation [7]. The findings of Yıldırım et al. [41] support this finding and state that OECD economies should support environmental innovation since it can reduce environmental pollution.

The IEA [79] illustrates how research and development has led to improvements in energy efficiency. These innovations have enabled businesses and households to lower their carbon footprint while increasing their productivity. In addition, implementing modern grid management systems has enabled better integration of renewable energy sources, which has optimized energy use and minimized environmental implications, as observed by the OECD. The OECD Environmental Outlook highlights that these technologies contribute to reaching climate targets and position OECD economies at the forefront of the worldwide transition to a low-carbon energy sector. Given these concerns, continuing support for R&D projects in energy technology is essential for maintaining the dual goals of economic development and environmental preservation among the nations that comprise the OECD [7]. Previous research has yielded comparable results to our findings, as demonstrated by Sun and Razzaq [44] in their study of OECD countries, Ahmed et al. [80] in G7, Sun et al. [81] in their examination of polluted economies, and Chen et al. [3] in their analysis of China. This study challenges the conclusions of Shao et al. [82] in short run, Ahmed et al. [83] for their study of USA, Mourshed and Quddus [84] for the European nations and Koçak and Ulucak [85] for the OECD.

Table 6 shows SFGI research results, indicating a significant correlation between the state fragility index and SDI in both short and long-term perspectives across studied OECD economies. Even a 1% rise in the SFGI can cause to decline of about 0.128% and 0.065% in the Short-term and Long-term SDI, respectively, across OECD countries. Consequently, the unfavourable impact of SFGI on sustainable development is caused by the significant influence that weakened political institutions have on SDI. The outcome aligns with the results of Saba and Ngepah [86] for Africa and Common Market for Eastern and Southern Africa where they found that state fragility is harmful for growth. However, our result disagrees with their result for Arab Maghreb union, East African community, Economic Community of West African States (ECOWAS) and Southern African Development Community where authors found no impact or positive impact of state fragility on GDP. Furthermore, Chuku and Onye [87] confirmed that higher state fragility entails greater macroeconomic crisis and volatility for the African nations.

Table 6 highlights the short-run results that depict that FIN is positively correlated with SDI in selected OECD economies in the long as well as short run. It shows that a % increase in FIN significantly enhances the SDI by approximately 0.240% and 0.415% in the long and short run in OECD economies, respectively. This study matches with the outcome of Charffeddine and Khediri [88] where the authors found that FIN provides easy access to people towards clean energy, resulting in enhanced environmental sustainability. However, the study finding is not in line with Li et al. [89] as they found no significant effect of FIN on economic sustainability for ECOWAS nations. This study also challenges the finding of Islam et al. [90], where they demonstrated that financial development adversely influences sustainable development in ASEAN countries. Fakher et al. [91] also found that financial development harms environmental quality. Consequently, this result suggests that OECD countries need to transform their financial institutions towards green finance centric institutions so as to sustain their development. Currently, these countries have a high concentration towards investment in fossil fuel energy projects. However, the results of this study argue for more green finance-based projects in these economies.

The short-term and long-term impacts of FDI are illustrated in Table 6. Moreover, the results of CS-ARDL for FDI depict that increased FDI substantially promotes SDI. The SDI in OECD economies experienced a short-term increase of 0.077% and a long-term increase of 0.049%, with every 1% rise in the FDI level. The outcome is similar to past studies such as Borensztien et al. [92] in the case of OECD economies, and Lee [93] for G-20 economies. For African countries, Aust et al. [94] discovered that FDI boosts the agenda of SDG. However, Ofori et al. [95] has demonstrated that FDI is harmful for green development, contradicting our study. Ayamba et al. [96] has also shown that FDI does not influence environmental quality during the long run. Caetano et al. [97] additionally discovered that energy consumption improves via FDI, which in turn increases pollution and harms sustainable development of OECD nations.

### 4.4 Robustness check

A comprehensive investigation was carried out to bolster the dependability of our findings as part of establishing the validity of the extensiveness of our findings. Two methods were used to validate the results of CS-ARDL. First, CCE-GMM by Neal [24] was used to handle the reverse causality between SDI and ENIN. Secondly, CCE-MG estimator of Pesaran [25] was used. The findings of this robustness assessment are demonstrated in Tables 8 and 9, in a manner that is both comprehensive and straightforward, and they demonstrate a significant alignment with the conclusions reached from our earlier CS-ARDL analysis. This coherence across approaches strengthens the legitimacy of our findings and instils greater trust in the associations that have been formed.

**Table 8:** Result of CCE-GMM.

D.lnSDI	Coef.	Std. Err.	P value
lnENIN	0.053637	0.023803	0.024
lnSFGI	-0.06528	0.022116	0.003
lnFIN	0.083144	0.038703	0.032
lnFDI	0.060216	0.027604	0.029
Constant	-0.00336	0.030135	0.911
Wald Chi2	25.49	-	0.000

Source: Authors’ own calculations

**Table 9:** Common Correlated Effects Mean Group (CCE-MG) estimator.

Variables	Coef.	Std. Err.	P value
lnENIN	0.047	0.018	0.01
lnFIN	0.061	0.021	0.004
lnFDI	0.412	0.238	0.084
lnSFGI	-0.083	0.033	0.013
Constant	-1.627	0.874	0.063
Wald chi2	16.28	-	0.0027
Root Mean Squared Error (sigma)		0.0345	

Source: Authors’ own calculations

The variable ENIN emerges as a robust factor, displaying a persistent and significant link with the Sustainable Development Index (SDI) in both methods. This is evidenced by the connection observed across both short-run and long-run. Because the results of the robustness check and the CS-ARDL analysis are congruent, this lends credence to the dependability of our prior observations.

In addition, the robustness check provides further insights by highlighting the significant roles that FDI and FIN play in the process of sculpting the landscape of sustainable development within the economies of the OECD. The fact that these variables have been endorsed repeatedly as crucial contributors highlights the significance of their role as credible and practical possibilities for encouraging sustainable developmental trajectories within the context of this discussion. Similarly, the linkage between SFGI and SDI underscores the consistency of our findings across different econometric approaches. The robustness check further underscores the inherent vulnerability that weakened political institutions and the dimensions of state fragility can introduce to the pursuit of sustainable development goals. This robustness check supports the notion that addressing state fragility is pivotal for fostering a more sustainable and resilient development trajectory.

## 5.0 Conclusion and policy implications

This study gaols to examine the role of energy technology innovation in the SD of 28 OECD countries. We select energy technology innovation as our focus variable proxied by the energy technology R&D budget. Sustainable development entails a development index as well as an ecological impact index. In addition to energy technology innovation, we consider several other control variables such as state fragility index, FIN and FDI. The state fragility index is taken to ensure whether a fragile state is prone to unsustainable development. To realize the above objective, we employ several econometric tests. For example, we first consider CSD, unit root test, and cointegration test. We used the CS-ARDL model for the long-run and short-run analysis. We considered the CCE-GMM by Neal [24] and the CCE-MG estimator by Pesaran [25] for robustness analysis.

The study findings provide valuable insights for policy recommendations. Since energy technology promotes sustainable development, governments of OECD countries should increase their energy technology budget. These countries should give incentives to the firms to encourage energy innovation. Specifically, credit availability should be easy, and the process of accessing credit should be smooth. Infrastructure reforms should be made to enhance energy innovation.

These countries under consideration must encourage partnerships among people and public and private enterprises. This will assist citizens to become aware of different energy innovation solutions. Also, it will help these energy technologies to be replicated for the advancement of the SDG agenda. Energy innovation solutions should also be incorporated into educational curriculums so that students become aware of these as well as the environmental welfare associated with these solutions. However, only these solutions are not enough as a proper business environment is also required to replicate these innovations. In order to make energy technology accessible, the OECD region must make the environment of business collusive. As a result, the transfer of technology can be much easier, and new companies will start their operations. However, fragility in the form of corruption can prevent the implementation of energy technology in these nations. Therefore, the OECD region must initiate legislative and regulatory framework in order to improve energy innovation. To avail the energy solutions, financial institutions such as banks have crucial roles to play. Specifically, they should roll out a discriminatory interest rate based on the firms’ carbon footprint. For example, In order to dissuade firms and industries from adopting fossil fuel energy solutions, it may be beneficial to impose higher interest rates on those with a larger carbon footprint. As a result, SDG 7, which advocates for cleaner energy will be realized. It will also help realize the goal of SDG 13 (Climate action) as different companies and organizations will be forced to implement cleaner energy solutions in their operations to decrease their environmental footprint.

Sustainable development can also be ensured by lowering activities that encourage state fragility. That is, governments at all levels should curtail corruption and reduce violence. The financial sector must be strengthened, and foreign direct investment should be directed towards clean energy innovation for SD. Since FDI exerts a positive influence on SD, these nations should attract clean foreign capital instead of dirty FDI flows. While relaxing regulations to attract foreign business is encouraged, stricter environmental regulations should also be put in place. Policy makers must regulate clean FDI inflows to foster the transfer of green technology and green energy resources. The foreign firms already operational in the OECD region must undertake initiatives to promote a sustainable environment and clean energy solutions. Undertaking these activities might result in some short-term losses. However, they can avail long-term sustainable solutions through these processes. The policymakers should provide higher restrictions on firms importing dirty technologies in order to foster the SDG agenda.

Due to data unavailability, this research only considered 28 countries of the OECD. Hence, future research may fully benefit from the prospective data availability of energy technology innovation. Future studies can also compare different forms of energy technology innovation and assess if they have similar impacts on sustainable development.

## Supporting information

S1 Graphical abstract (TIF)

## List of abbreviations

AI: Artificial Intelligence
AMG: Augmented Mean Group
ARDL: Autoregressive and Distributed Lag
ASEAN: Association of Southeast Asian Nations
BRCS: Brazil, Russia, India, China, South Africa
CADF: Cross-Section Augmented Dickey-Fuller
CCEMG: Common Correlated Effects Mean Group
CCUS: CO2, Capture, Usage, and Storage Technology
CIPS: Cross-Section Im-Pesaran
CO2: Carbon dioxide emission
COP: Conference of Parties
CS-ARDL: Cross-Sectionally Autoregressive and Distributed Lag
CSD: Cross-Sectional Dependency
DOLS: Dynamic Ordinary Least Square
ECM: Error Correction Model
ECOWAS: Economic Community of West African States
ENIN: Energy Innovation
EU: European Union
FDI: Foreign Direct Investment
FIN: Financial Development
FMOLS: Fully Modified Ordinary Least Square
GDP: Gross Domestic Product
GHG: Greenhouse Gases
GMM: Generalized Method of Moments
IEA: International Energy Agency
INSCR: Integrated Network for Societal Conflict Research
JRC: Joint Research Centre
LM: Lagrange Multiplier
MENA: Middle East and North Africa
OECD: Organisation for Economic Cooperation and Development
R&D: Research & Development
RES: Renewable Energy Supply
SDG: Sustainable Development Goal
SDI: Sustainable Development Index
SFGI: State Fragile Index
SD: Sustainable Development
TPES: Total Primary Energy Supply
UK: United Kingdom
USA: United States of America
WDI: World Development Indicators
