# Supplementary figures and images for: From invention to progress: Energy technology innovation and sustainable development in OECD economies
Source: PLoS One. 2025 Feb 13;20(2):e0310104. doi: 10.1371/journal.pone.0310104 (PMC11824976; doi:10.1371/journal.pone.0310104)

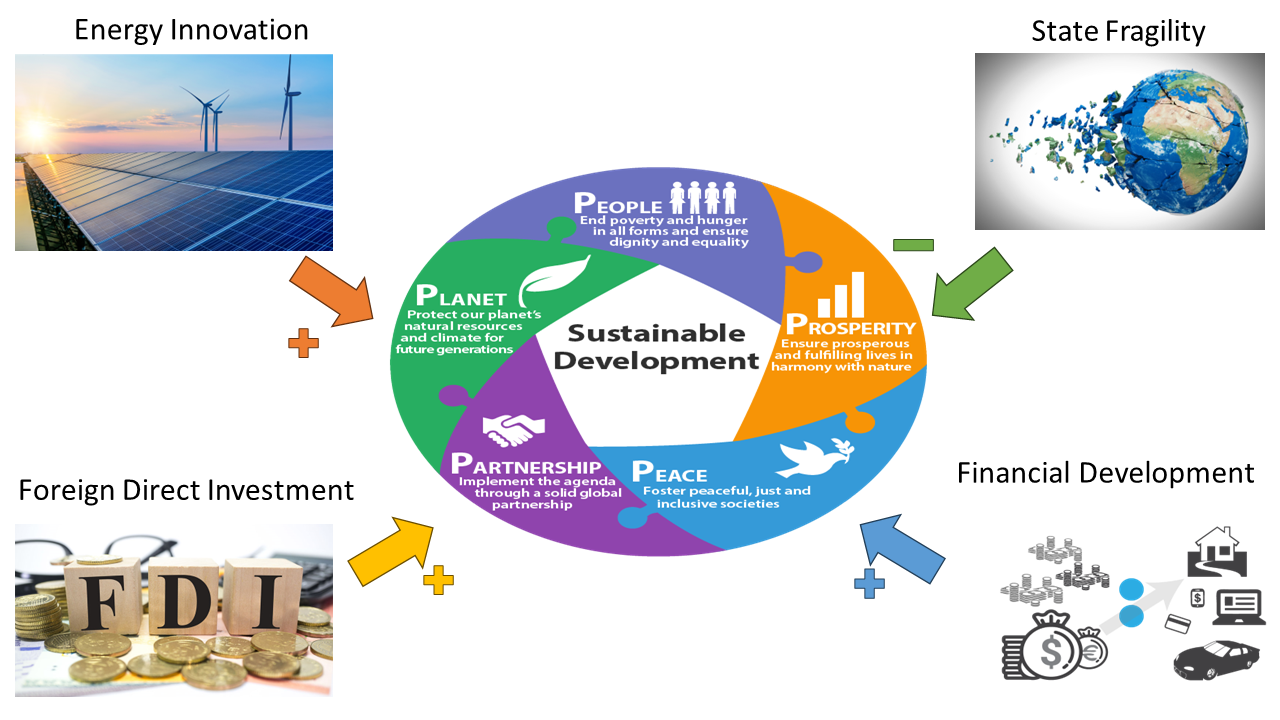

Supplement: S1 Graphical abstract — (TIF) [file pone.0310104.s001.tif]
